# Melanoma Tumour Vascularization and Tissue-Resident Endothelial Progenitor Cells

**DOI:** 10.3390/cancers14174216

**Published:** 2022-08-30

**Authors:** Ghazaleh Hashemi, James Dight, Kiarash Khosrotehrani, Laura Sormani

**Affiliations:** Experimental Dermatology Group, Dermatology Research Centre, The UQ Diamantina Institute, The University of Queensland, Woolloongabba, QLD 4102, Australia

**Keywords:** melanoma vascularization, vasculogenesis, endothelial progenitor cells, anti-angiogenic drugs

## Abstract

**Simple Summary:**

Melanoma is the most aggressive and potentially lethal form of skin cancer. Research over recent decades has highlighted the role of tumour vasculature in altering the metabolic function of cancer cells, infiltration of immune cells, and cancer cell dissemination. However, variations in the modes of vessel formation in melanoma have made this process difficult to target. In particular, the role of endothelial progenitor cells in melanoma vascularization-promoting vasculogenesis begins to be understood. Progenitor recruitment, vessel formation, and paracrine activity are among the steps contributing to tumour metastasis and affecting the impact of anti-angiogenic drugs, as detailed in this review.

**Abstract:**

The aggressiveness of solid cancers, such as melanoma, relies on their metastatic potential. It has become evident that this key cause of mortality is largely conferred by the tumour-associated stromal cells, especially endothelial cells. In addition to their essential role in the formation of the tumour vasculature, endothelial cells significantly contribute to the establishment of the tumour microenvironment, thus enabling the dissemination of cancer cells. Melanoma tumour vascularization occurs through diverse biological processes. Vasculogenesis is the formation of de novo blood vessels from endothelial progenitor cells (EPCs), and recent research has shown the role of EPCs in melanoma tumour vascularization. A more detailed understanding of the complex role of EPCs and how they contribute to the abnormal vessel structures in tumours is of importance. Moreover, anti-angiogenic drugs have a limited effect on melanoma tumour vascularization, and the role of these drugs on EPCs remains to be clarified. Overall, targeting cancer vasculature remains a challenge, and the role of anti-angiogenic drugs and combination therapies in melanoma, a focus of this review, is an area of extensive exploration.

## 1. Introduction

Melanoma is one the most aggressive form of skin cancer emanating from its pigment-producing cells, melanocytes [1]. In the past few decades, melanoma incidents have gradually risen, and around 65% of skin-related deaths are caused by cutaneous melanoma [2]. Sun exposure is one of the major risk factors and leads to a genetic signature that is the feature of melanoma. Melanoma has the highest mutation load among all cancers. BRAF (around 50%) and NRAS (15–30%) are the most common mutations in melanoma, resulting in the activation of the MAPK pathway [3]. Sporadic melanomas (~90%) are caused by the high prevalence low risk alleles, which emphasise the causative role of environmental factors on the progression of melanoma. For instance, studies demonstrated that melanocortin-1 receptor, a highly polymorphic gene, is associated with red hair and increased risk of melanoma via pigmentary and non-pigmentary pathways [4]. Gene expressional profiling of patient signatures may assist in the adjuvant systemic therapy or targeted therapy. Early surgical excision of the primary tumour with adequate margins is the most effective treatment, although melanoma cells can disseminate early, prior to dissection [5]. Cancer cell dissemination is the leading cause of melanoma, related mortality. In advanced disease or the adjuvant setting, targeted therapy with BRAF and MEK inhibitors or immune checkpoint inhibitors (ICIs) have advanced patient survival and outcomes [6]. However, the efficacy and effectiveness of these treatments are not consistent for all patients [7], prompting the need for new approaches. Melanoma cells modulate the tissue environment, in particular, immune cells, extra cellular matrix, and endothelial cells. The acquisition of vascularization is commonplace for melanoma [8]. Studies using doppler ultrasound have detected increased blood flow in melanoma tumours thicker than 0.9 mm. Patients with recurrent tumours or metastases present more vascularized primary tumours, compared to those free of recurrence [9,10].

During development and adulthood, endothelial cells form a network of blood vessels to facilitate the exchange of oxygen, deliver nutrients and immune cells, and discard waste from organs. Blood vessel formation is crucial for regeneration and tissue growth. Paradoxically, this network enables the growth of tumours and their spread, allowing an escape route toward metastatic spread [11,12]. Unlike physiological situations, tumours have various modes of acquiring vessel formation, with a broad impact on the tumour microenvironment and progression. The mechanism of sprouting angiogenesis, whereby vessels develop from the pre-existing vascular network, is by far the most well-studied and targeted in cancer therapy. Many anti-angiogenic strategies, mainly VEGF blockers, have attempted to reduce tumour vascularization in melanoma, although it seems to be ineffective, as monotherapy and only a subset of patients benefited from these treatments [13]. The limited success of anti-angiogenic approaches, as a single agent, can be justified by the variety of vessel formation modalities, such as vascular co-option, a process where cancer cells hijack nearby existing blood vessels. The other form of vessel formation is the vascular mimicry, which takes place independently of angiogenesis; cancer cells obtain an endothelial-like phenotype and form vascular networks without endothelial cells. The EPCs recruitment and de novo blood vessel formation, a process called vasculogenesis, also contribute to the vessel formation. Emerging evidence has pointed to the role and potential of EPCs and their therapeutic target. However, the mechanisms and origin of the endothelial cells that might contribute to the tumour neo-vessels are controversial. Here, we review the various modes of tumour vascularization and role of EPCs in melanoma vascularization. Moreover, we report the impact of some of the recent clinical trials of anti-angiogenic drugs in melanoma treatments.

## 2. How Do Tumour Blood Vessels Form in Melanoma?

**Figure 1:**
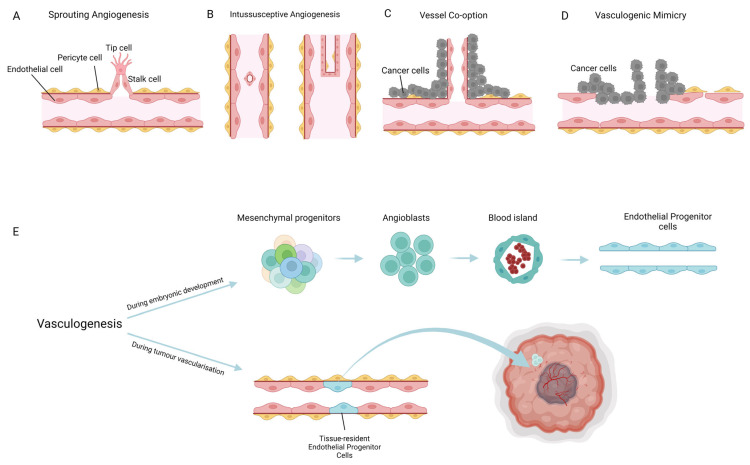
Mechanisms of blood vessel formation in melanoma. (**A**) Sprouting angiogenesis: formation of blood vessels from existing blood vessels. (**B**) Intussusceptive angiogenesis: formation of blood vessels by splitting a blood vessel into two. (**C**) Vessel co-option: melanoma cells relocate along the abluminal surface of the vessels. (**D**) Vasculogenic mimicry: melanoma cells remodel and form looping patterns and channels mimicking the vasculature. (**E**) Vasculogenesis: formation of de novo blood vessels from EPCs. Vasculogenesis occurs during embryonic development from angioblasts and during adulthood and pathological conditions, such as tumour vascularization, from tissue-resident endothelial progenitor cells. Created with BioRender.com.

Tumour vascular formation in cancer has been the centre of attention since the 1970s. Endothelial cells can be molecularly targeted to prevent tumour growth, metastasis, and blood vessel formation, which has been of great interest for therapeutic purposes [14]. The work by Folkman initiated our appreciation of the importance of vascularization in the tumour. Folkman and colleagues demonstrated endothelial cells can be stimulated by cancer cells and proliferate and contribute to tumour growth as the result. They observed that highly vascularised tumours grow rapidly, compared to poorly vascularised tumours [15]. Several modes of vessel formation have been described in tumours: sprouting angiogenesis, intussusceptive angiogenesis, vessel co-option, vasculogenic mimicry, and vasculogenesis (Figure 1). Here, we emphasise the molecular mechanisms of each form of blood vessel formation to better understand their role in tumour growth.

### 2.1. Sprouting Angiogenesis

The term angiogenesis was coined by Dr John Hunter for the first time in 1787 [16,17]. At the beginning of the 20th century, Edwin E. Goldmann focused on the importance of the vascular system in the growth of tumours and circulation of malignant cells and reported the multiplication of blood vessels in a tumour [18]. Soon after that, other studies suggested that factors secreted by tumour cells were responsible for the development of vessels in the tumour. Since these early descriptions, much progress has been made in the detailed molecular mechanisms driving angiogenesis.

The formation of new blood vessels is an essential process in tissue development, regeneration, and repair. Angiogenesis is the process of blood vessel formation from a pre-existing vascular network [19]. Endothelial cells, pericytes, and smooth muscle cells share a basement membrane, which comprise extracellular matrix proteins, mainly laminin, collagen types IV, XV, and XVIII, fibronectin, nidogen, and the heparin sulfate proteoglycan perlecan [20]. These extracellular matrix proteins support sprouting angiogenesis, endothelial cell adhesion, and migration [21]. Upon release of angiogenic signals, such as VEGF, angiopoietin 2 (ANG2), fibroblast growth factors (FGFs), chemokines released by cancer cells, hypoxia, or injury, the basement membrane is degraded by membrane-type matrix metalloproteases (MMPs), MMP3, MMP9, and MMP10. The combination of these MMPs guides the invasion and migration of endothelial cells during sprouting angiogenesis [21,22]. Pericytes first detach from the basement membrane after stimulation by ANG2, and endothelial cells loosen their junctions [23]. The sprouting angiogenesis is regulated by an interplay between the endothelial tip and stalk cells. Tip cells are the migratory leading endothelial cell and block the neighbouring cells, stalk cells, from adopting the same fate. The stalk cells follow the tip cells to elongate and develop sprouting vessels (Figure 1A). The specification of tip and stalk cells is dependent on Notch signalling, which controls vascular stabilisation, cell elongation, proliferation, and differentiation [24,25]. In stalk cells, there is a high activity of canonical notch signalling, in contrast to a low level of notch signalling in tip cells [26]. Delta-like ligand 4 (DLL4) is expressed by tip cells at a high level, compared to Notch ligand Jagged1 by stalk cells. VEGF stimulates the tip cells and formation of filopodia through VEGFR2 activation, which results in the enhancement of DLL4 in the tip cells [27]. The binding of DLL4 to Notch1 in the neighbouring endothelial cells activates Notch signalling and prevents the tip cell behaviour in those cells by upregulation of VEGFR1 (acting mostly as a decoy receptor for VEGF). Moreover, the activation of Notch1 by DLL4 downregulates VEGFR2, VEGFR3, and Neuropilin1, a process that is called “lateral inhibition”. The binding of DLL4 to Notch1 involves the cleavage of Notch receptors and release of the notch intracellular domain (NICD). Translocation of NICD to the nucleus is accompanied by binding to recombination signal binding protein for immunoglobulin Kappa J (RBPJ) and activation of SMAD6 [28]. Notch signalling is also responsible for endothelial branching, in response to bone morphogenic protein (BMP) signalling. BMP2 and BMP6 promote lateral vessel branching by increasing the level of nuclear pSMAD1/5 in the tip cells and suppression of pSMAD1/5 in the stalk cells [29]. Mathematical frameworks demonstrated that Jagged-Delta asymmetry in Notch signalling gives rise to a novel hybrid state, i.e., sender/receiver. The hybrid cells have both the Notch receptor and ligand, allowing the Notch–Delta–Jagged signalling to decide their fate [30]. Another study by Koon et al., showed the intermediate tip and stalk cells by immunofluorescent staining of tip cell markers [31]. This hybrid of tip/stalk cells might explain the existence of tip-like filopodia across the vessel lumen in the tumour and provide valuable information regarding tumour stroma cross-talk [32].

Sprouting angiogenesis was studied by Warren et al., for the first time by melanoma transplantation in the cheek pouch of the Syrian hamster. They observed capillary invasion and development as a regular sequence [33]. Tumour-derived exosomes released by melanoma cells are loaded by pro-angiogenic factors, such as VEGFA and MMPs. Serum analysis of human melanoma cells demonstrated the high expression of VEGFA, which could be utilised to monitor different melanoma stages [34]. Moreover, VEGFA expression is correlated with the progression of cutaneous melanoma [35]. Melanoma exosomes interact with endothelial cells by moving between them and influencing endothelial tubule morphology [36]. In a study by Hood et al., it was revealed exosomes released from B16 melanoma cells engaged with murine 2F–2B endothelial cells and accelerated the rate of endothelial cells’ proliferation and sprouting. Furthermore, B16 melanoma exosomes increased proangiogenic cytokines, such as FGF, G-CSF, VEGF, TGFα, and TNFα, with increasing exosome dosing [37]. Human melanoma cells’ exosomes also demonstrated similar results on endothelial colony-forming cells and human microvascular endothelial cells, where urokinase-type plasminogen activator receptor (uPAR) released by exosomes induced proangiogenic activity [38]. uPAR is considered a potential prognostic marker, and it is upregulated in some human melanoma cells [39]. Analyses of 255 human nodular melanoma tumours and 78 matched loco-regional metastases demonstrated a high uPAR expression and high vascular proliferation index. This was associated with increased tumour thickness, tumour ulceration, and reduced overall survival of patients [40]. These studies showed the importance of sprouting angiogenesis in melanoma. The angiogenic factors secreted by melanoma cells are significantly correlated with tumour progression, tumour invasion, and the overall survival rate of patients [41]. However, targeting the VEGF pathway alone is not sufficient, as there are compensatory pathways that melanoma tumours choose, such as alternative modes of vessel formation.

### 2.2. Intussusceptive Angiogenesis

Most of the literature has mainly focused on sprouting angiogenesis as the main mechanism of angiogenesis. An alternative mechanism of melanoma vascularization is intussusceptive angiogenesis. In this form of vascularization, the capillary wall expands into the lumen of a vessel and splits it into two new vessels. This mode of angiogenesis was first recognised in rat lungs in the late 20th century [42]. During postnatal development, parenchymal septa of the lung contain two capillary layers. Using scanning electron microscopy, Caduff et al., observed the fusion of the two capillary networks, which led to the mature vascular network [43]. Intussusceptive angiogenesis initiates with the creation of an area of contact between opposite endothelial cell walls, followed by the establishment of intercellular endothelial junctions and formation of a transcapillary pore. As the pore widens, myofibroblast and pericytes invade the pillar that deposits the extracellular matrix into the capillaries. In the last step, the capillaries enlarge in size to form a mesh [44] (Figure 1B).

The molecular mechanism of intussusceptive angiogenesis is less studied, however VEGF has been shown to be involved in this form of vascularization. For the first time, Ribatti and colleagues observed a strong expression of VEGF in human primary melanoma tumours, which correlated with increased microvascular density, vessel diameter, and intussusceptive microvascular growth [45]. Esteban and colleagues identified membrane type 1 (MT1)-MMP from endothelial cells cleaved the thrombospondin 1 (TSP1) and promoted nitric oxide production, vasodilation, and intussusceptive angiogenesis in colitis. Targeting the MT1-MMP/TSP1 pathway via the monoclonal antibody has the translational potential to reduce intussusceptive angiogenesis [46]. In another study by Pandita et al., it was demonstrated that intravascular pillars are present in metastatic human melanoma tumours, and the expression of MMP9 was higher in human metastases, compared to the patient-derived melanoma xenografts in mice. In vitro MMP blockade inhibited the formation of pillars and MMP blockade could be utilised as an anti-angiogenic drug with the capacity to reduce metastasis in melanoma patients, suggesting the importance of this mode of angiogenesis [47]. Intussusceptive angiogenesis may contribute to the progression of melanoma, although future studies are required to investigate the molecular mechanism of intussusceptive angiogenesis further.

### 2.3. Vessel Co-Option

Tumours can hijack pre-existing blood vessels from surrounding non-malignant tissues in order to gain survival benefit, growth, and metastasis, which is known as vessel co-option [48]. This non-angiogenic process was explored by Pezzella and colleagues in the primary stage I non-small cell lung carcinoma [49,50]. Cancer cells relocate along the abluminal surface of the vessels or invade the normal tissue between the vessels to resolve their metabolic needs [48] (Figure 1C). The morphology of vessels is preserved, such that the overall structure and branching pattern of the vessels are generally similar to that of the non-malignant tissue [51]. The rate of endothelial cell proliferation in the vessel co-option is limited and reduced, compared to the rate of endothelial proliferation in sprouting angiogenesis. A recent single-cell RNA sequencing of renal adenocarcinoma lung metastasis demonstrated that the transcriptome of normal endothelial cells and co-opted tumour endothelial cells are alike [52]. Furthermore, they observed macrophages might facilitate the migration of cancer cells to co-opt vessels via matrix-remodelling and keep the endothelial cells quiescent [52]. Preclinical data in human and murine melanoma tumours indicate the incorporation of the peritumoural vascular network into the growing tumour [53]. Vessel co-option is mainly observed in the metastatic models of melanoma. Human melanoma cells, upon injection into the brain of nude mice, start to migrate along the abluminal surface of microvascular channels and spread to other parts of the brain over time [54]. Melanoma cells can move along the abluminal surface of the blood vessels due to their neural crest-derived migratory origin [55].

Lugassy and Barnhill introduced the field of extravascular migratory metastasis (EVMM) in melanoma [56]. In EVMM, angiotropic melanoma cells are defined as cells that are associated closely with the endothelial cells in the vasculature, such as pericytes. In this process, tumour cells spread along the external vascular surface and metastasise to distant organs without intravasation. Angiotropism is the phenomenon that is used to describe microscopic pericyte-like growth [57]. Preclinical data from human and murine samples indicate that melanoma tumours grow by incorporating the tumour-induced vascular plexus in the peri-tumoural connective tissue [53]. Melanoma patients exhibit either lymph node metastasis or macroscopic or microscopic satellites. Melanoma samples with the microscopic satellite have been demonstrated to have more angiotropism or pericyte mimicry [58]. Satellites are defined as nodules or tumours associated with intralymphatic metastasis. Microscopic satellites are greater than 0.05 mm [59]. Furthermore, angiotropism in the primary cutaneous melanoma correlates with brain [60], liver [61], and lung [62] metastasis. Anti-angiogenic blocking antibodies against VEGFA prevent angiogenesis, but not vessel co-option and melanoma brain metastasis [63]. Better understanding of the molecular mechanisms of vessel co-option has translational potential.

### 2.4. Vasculogenic Mimicry

In 1999, Maniotis and co-workers initiated the concept of vascular mimicry in human uveal melanoma. The highly metastatic melanoma cells remodel and form looping patterns and channels that mimic the vasculature [64] (Figure 1D). Plastic tumour cells develop a fluid-conducting network, without the involvement of endothelial cells. In this process, tumour cells mimic the gene expression and phenotype of endothelial cells [65]. Two types of vascular mimicry have been observed in tumours: the patterned matrix and tubular types. The patterned matrix type has no resemblance to blood vessels and comprises matrix proteins, such as collagen IV [66] and VI, laminin [67], and heparan sulfate proteoglycan [68]. The tubular type comprises cancer cells that mimic endothelial cells and develop a tube-like network [69]. Radnot et al., detected vascular channels in the human uveal melanoma, which were lined with both endothelial and melanoma cells. In most cases, the disruption of the vessel wall and release of the melanoma cells into the lumen of the vasculature were observed [70].

Melanoma cells’ plasticity is the foundation of vasculogenic mimicry. In the early collaborative study at the National Human Genome Research Institute (NHGRI), microarray analysis of uveal melanoma cells revealed the expression of Tie1, epithelial cell kinase, and some other genes that facilitate the formation of blood vessels, such as connective tissue growth factor, collagen I and VI, and fibronectin [71]. Various studies reported that highly metastatic melanoma cells express VE-cadherin [72,73], Tie1 [72], CD31 [74], and vimentin [75]. In a recent study by Martini et al., CD36, a cell surface fatty-acid receptor has been demonstrated to advance vascular mimicry in human melanoma tumours. Of importance, they confirmed the expression of VE-cadherin on the endothelial cells, but not on any of the melanoma cell lines [76]. Other studies reported the negative expression of CD31 in the human melanoma cell lines, contradicting other studies [72,77]. These pivotal studies prompted future investigations into the mechanism of vascular mimicry. VEGF controls vascular mimicry via PI3/AKT signalling pathway. VEGF knockout in the OCM-1 choroidal melanoma cell line reduced p-AKT, and the expression of MT1-MMP, MMP2, and MMP9, which impaired vasculogenic mimicry [78]. A recent study investigated how VEGF/PDGF pathway controls vascular mimicry in a Wnt5/β catenin manner. Dual knockdown of VEGF/PDGF inhibited vascular mimicry and resulted in cell apoptosis [79]. As discussed here, there remains controversy in the gene expression and specific markers for distinguishing the endothelial and melanoma cells that contribute to vasculogenic mimicry.

### 2.5. Vasculogenesis

Vasculogenesis is defined by the formation of de novo blood vessels in an avascular site. It is derived from mesodermal endothelial precursors, called angioblasts, in the developing embryo (Figure 1E). During embryonic life, vasculogenesis starts with mesoderm formation and alteration in paracrine signalling, which results in the migration of mesodermal cells. This migration leads to the formation of blood islands, consisting of hemopoietic cells surrounded by angioblasts. Signal transduction of FGF and other growth factors [80] lead to the differentiation of angioblasts towards EPCs. Further differentiation of EPCs allows endothelial cells to mature from the primary capillary plexus [81]. It was believed that vasculogenesis occurs only during development. However, Asahara et al., reported equivalent observations postnatally [82]. In the next section, a deeper review of the characteristic of EPCs and their contribution to melanoma vascularization is explored.

## 3. Role of Endothelial Progenitor Cells in Tumour Vascularization

The function of postnatal EPCs in tumour vascularization and metastasis has been the focus of many studies. However, identifying the origin of these cells presents new challenges. In 1997, putative EPCs or angioblasts were isolated from human peripheral blood, and these cells had the potential to give rise to endothelial cells, with the capacity to augment vascularization in ischemic models. These cells were isolated based on the surface expression of CD34^+^ and VEGFR2^+^ (Flk-1). These circulating endothelial cells displayed high levels of endothelial markers, such as platelet endothelial cell adhesion molecule 1, Tie2, endothelial nitric oxide synthesis (eNOS), and low levels of hematopoietic markers in culture [82,83]. In these initial descriptions, primarily EPCs derived from bone marrow, expressed CD133 (a hematopoietic stem cell marker), CD34, and VEGFR2. After 4–7 weeks in culture, the expression of CD133 was lost; they maintained weak CD34 and VEGFR2 and gained the expression of CD31, VE-cadherin, and von Willebrand factor (vWF) [84,85]. Subsequent studies established evidence that many of these circulating cells were, in fact, hematopoietic adopting the surface markers of other cells [86] but, did not incorporate into blood vessels in ischemic tissue [87] or tumours [88]. Particularly, myeloid progenitor cells derived from bone marrow were more likely to adopt this endothelial cell-like phenotype [89,90]. Here, our focus is on postnatal EPCs and their contribution to vascularization.

**Figure 2:**
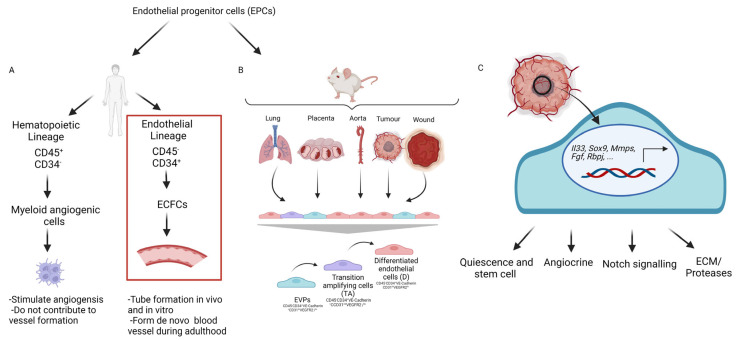
The endovascular progenitor cells in human and mouse. (**A**) According to phenotypic lineage, there are two distinct population of EPCs, the myeloid angiogenic cells and ECFCs. ECFCs do not have hematopoietic origin, although can form de novo blood vessels and colonies, while the myeloid angiogenic cells stimulate angiogenesis and do not contribute to vessel formation. (**B**) Endovascular progenitor cells (EVPs) have been identified in the vessel wall of various organ beds in the murine model. EVPs can give rise to transition amplifying (TA) cells and mature differentiated (D) endothelial cells. (**C**) EVPs have been also characterised in the murine model of melanoma. EVPs express stem cell, angiocrine, notch signalling, and extracellular matrix (ECM)/proteases genes. Created with BioRender.com.

In recognition of these difficulties in lineage and nomenclature, a new consensus was recently adopted. Medina et al., characterised two distinct populations of EPCs, based on their phenotype in humans: the early EPCs, called myeloid angiogenic cells, that have a hematopoietic origin and late EPCs, called endothelial colony-forming cells (ECFCs), that possess endothelial phenotype. These populations were named: hematopoietic EPCs and non-hematopoietic EPCs [91] or ECFCs [92] (Figure 2A). ECFCs can maintain their proliferation and contribute to blood vessel formation [93]. An array of studies demonstrated that tissue-resident EPCs, which originate from the vascular beds of tissues and not the circulating cells, contribute to neovascularization, and these cells do not have bone marrow origin [94,95]. Employing functional assessment and colony-forming potential in cells from the umbilical cord blood, Ingram et al., identified a hierarchy within the endothelium. These cells expressed CD31, CD105, CD146, CD141, CD144, Flk-1, and vWF and did not express CD14 and CD45. They formed capillary-like structures in the Matrigel and could be expanded in culture. Based on single-cell and colony formation assay, they introduced an EPC hierarchy. The high proliferative, potential-endothelial, colony-forming cells contained the self-renewing fraction of ECFCs, as opposed to endothelial cell clusters that had no proliferative or self-renewal ability after 14 days from a single cell. Mature and differentiated cells lost their division potential and did not grow [96]. Aside from cord blood, ECFCs with similar potency can be isolated from other tissues, such as the placenta, with similar behaviour and function [97]. This work has formed the basis for EPCs to be classified by their self-renewal and vessel-forming ability, distinguishing myeloid angiogenic cells [98].

Fang et al., isolated endothelial progenitor cells from the murine model, with the characteristic of lin^−^CD31^+^CD105^+^ from adult digested lung tissue. These endothelial colony-forming cells gave rise to millions of daughter cells in vitro. They demonstrated high proliferative sorted cells expressed Sca-1 and c-Kit. This high proliferation of endothelial cells was detected in the lung, liver, subcutaneous tissues, kidney, and B16 melanoma tumours. The vascular endothelial cells were obtained from B16 melanoma vessels, with the potential to generate functional blood vessels in vivo, suggesting these cells had progenitor activity [99]. Although a distinct murine equivalent to ECFCs has not been identified, several studies have proposed progenitor populations that recapitulate key functional characteristics of ECFCs. This has provided an insight into the importance of endothelial progenitors in tumour vascularization.

A distinct definition of endothelial progenitor cells and endothelial hierarchy in murine tissues was proposed by Patel et al., in 2017, thus leading to the discovery of endovascular progenitor (EVP) cells. EVPs have a monopotent capacity to form vessels in an ischemic model similar to human EPCs. EVP cells were first identified in vivo by flow cytometry in situations of skin wound healing as CD45^−^CD34^+^VE-cadherin^+^CD31^l^°VEGFR2^−^/^l^°. In RNA sequencing analysis, EVPs expressed matrix metalloprotease genes (*Mmp2*, *Mmp3*, *Mmp14*, *Mmp19, and Mmp23*) and growth factor receptors, important for cell mobility. Growth factor genes, such as *Egfr*, *Pdgfrα*, and *Pdgfrβ*, as well as genes that are important for stemness, such as *Il33* and *Sox9*, were upregulated. Using a variety of methods, such as colony-forming assays, transplantation, and lineage tracing, it was shown that EVPs give rise to transit-amplifying (TA) and differentiated endothelial (D) cells, in the context of wound healing. Similar populations could be observed in the aorta, lung, placenta, and tumour environment [100] (Figure 2B). Furthermore, EVPs were characterised via single-cell RNA sequencing in the aorta, with similar gene expression profiles as those identified in bulk RNA sequencing [101]. The unsupervised hierarchical clustering analysis revealed two main populations: a mature, differentiated endothelial cell overexpressing *Sox17*, *Cd36*, *Fabp4*, and *Cldn5* and second, more mesenchymal, population corresponding to EVPs [101].

To examine the role of EVPs in melanoma tumour vascularization, with the aid of lineage tracing, Donovan et al., reported the infiltration of EVPs in melanoma primary tumours as early as 3 days after tumour inoculation [102]. EVPs were shown to migrate as single cells in early tumours and, over time, constituted vessels. The EVPs expressed *Sox18* and contributed mostly to both arterial and venous capillaries in pathological conditions. In a previous study, it was shown that *Sox18* was important in EVP progression to transit-amplifying and differentiated cells during wound revascularization [100]. *Sox18* regulates the lymphatic endothelial specification during development [103]. Its expression is usually lost in adulthood, although it is re-expressed during pathological conditions, including the murine model of melanoma [104]. Partial loss of *Sox18* has been reported to reduce the metastatic progression of melanoma [104], and inhibitors have been successfully employed to reduce metastases in preclinical breast cancer models, suggesting that EVP function might be important in tumour vascularization [105].

To better understand the function of EVPs in melanoma tumours, RNA sequencing of endothelial cells in the primary B16F0 melanoma tumour was carried out by Denovan et al. [102]. It was revealed that EVPs in the tumour share a molecular signature with the aorta. Stem cell genes (*Nfatc4*, *IL33* and *Cdkn1c*, *Aldh1a1*, and *Sox9*), proteases/ECM genes (*Mmp2*, *Mmp9*, *Mmp11*, *Mmp19*, *Col3a1*, *Col1a2*, and *Has1*), angiocrine genes (*Wnt*, *Fgf*, *Hgf*, *Vegfd*, *Vegfa, Pdgfc*, and *Tgfβ3*), and Notch signalling (*Notch3*, *Notch2*, *Rbpj, and Heyl*) were upregulated in EVPs, compared to differentiated endothelial cells in the tumour [102] (Figure 2C). An important characteristic of EVPs is the low level of VEGFR2 expression. Interestingly, EVP numbers were not affected when anti-VEGFA treatment was used to reduce melanoma vascularization. This study further showed that EVPs were important in the metastatic process. Indeed, the loss of Notch signalling through conditional deletion of *Rbpj* in the endothelium resulted in the complete depletion of EVPs and prevented metastases in a highly aggressive murine melanoma model [102]. This was supported by co-transplantation studies, where larger tumours developed when melanoma cells were delivered with EVPs, compared to differentiated endothelial cells [102]. Although the exact mechanisms by which EVPs exert their pro-metastatic effect are unclear, their angiocrine activity may affect tumour growth and metastasis.

## 4. How Do Endothelial Cells Reshape the Tumour Microenvironment?

Tumours are intricate environments defined by spatiotemporal communication between multiple cell types [106]. The primary tumour microenvironment consists of multiple cell types that communicate with each other and tumour cells via ligand-receptor interactions, cancer cell-derived exosomes, microRNAs, cytokines, chemokines, and growth factors [107]. The dynamic communication and paracrine activities of cancer cells with their microenvironment are crucial to the growth and metastasis of the tumour [108]. In this section, we elaborate on the role of EPCs, immune cells, and cancer-associated fibroblast (CAFs) in the tumour microenvironment.

Studies demonstrated that EPCs that are recruited into the tumour microenvironment, contribute to vascular network formation, and have a unique gene expressional profile [102]. The recruitment of EPCs is dependent on secreted factors by melanoma cells, immune cells, and chemokines in the tumour microenvironment. Laurenzana et al., demonstrated that ECFCs have translational relevance and can be used as a therapeutic agent [109]. The uPA and its receptor (uPAR) contribute to the progression, vascularization, and metastasis of melanoma. Furthermore, ECFCs can augment the invasiveness of melanoma cells in vitro [109]. In this study, MMP12 exhibited a different characteristic, compared to other MMP family members, in endothelial cells. MMP12 activation in ECFCs degraded uPAR and abolished angiogenesis and tumour growth in melanoma [109]. Moreover, they showed that the recruitment of ECFCs into the tumour microenvironment is dependent on the CXCR4/SDF1 pathway. In another study, it was revealed that MMP12-deficient mice accumulated myeloid-derived suppressor cells, which contributed to tumour growth [110]. There is a correlation between the MMP12, monocyte-derived macrophages, and endoglin expressed by endothelial cells in inflammation [111]. Studies demonstrate that endothelial cells have an important role in regulating the tumour microenvironment.

In solid tumours, the expansion of blood vessels associated with HIF1α, and the upregulation of the VEGF pathway leads to malformed and malfunctioning angiogenesis. This induces immunosuppressive characteristics such as T cell exhaustion and apoptosis [112]. Tumour endothelial cells and vasculature are the initial path to delivering immune cells to the tumour. In recent years, single-cell sequencing has provided an overview of the heterogeneity and diversity of the tumour microenvironment. Single-cell sequencing analysis of six treatment-naïve syngeneic mouse tumours, including melanoma, indicated cell–cell interactions in the tumour microenvironment [113]. The ECM-related interaction with endothelial cells and CAFs correlated with tumour growth. The expression of collagens by endothelial cells and CAFs facilitated the binding of these cells to CD93or integrin receptors on tumour cells [113]. CD93 is highly expressed in tumour endothelial cells in the B16 melanoma model [114]. The treatment of the B16 tumour with anti-CD93 antibody normalised tumour vasculature and suppressed tumour growth [114]. During tumour vessel normalisation, the pro- and anti-angiogenic factors, such as VEGFA and ANG2, come to a balance—pericyte coverage and perfusion increase and hypoxia decreases. Moreover, tumours switch from an immunosuppressive microenvironment to an immunosupportive microenvironment [115]. IGFBP7 is a ligand for CD93, and it is upregulated in tumour endothelial cells. Inhibition of the IGFBP7/CD93 pathway enhanced chemotherapy effectiveness, reduced hypoxia, and promoted drug delivery. Moreover, CD4^+^ T, CD8^+^ T, and natural killer (NK) cells were increased in tumours treated with CD93 inhibitor [114]. In another study, a deeper analysis of single-cell sequencing of lung, colorectal, and ovarian cancer endothelial cells revealed five subclusters of endothelial cells in these tumours [116]. Tip cells, high endothelial venules, venous endothelial cells, capillary, arterial, and lymphatic endothelial cells were identified. Clustering of endothelial cells after canonical correlation analysis showed four of these clusters were derived from normal tissue. Tip endothelial cells resided only in tumours, and high endothelial venules were more frequent in tumour tissues. The tumour endothelial cells expressed PLVAP and IGFBP7, and oxidative phosphorylation, glycolysis, and gluconeogenesis were upregulated in the metabolic activity analysis [116]. Tumour endothelial cells have the potential to induce an immune response in melanoma tumours. The stimulator of interferon (IFN) genes (STING), which binds strongly to cGAMP, is secreted by endothelial cells in the tumour microenvironment [117]. Demaria et al., showed that intratumoural injection of cGAMP into mice engrafted with B16F10 melanoma cells promoted CD8^+^ T cell infiltration in wild-type mice, compared to STING deficient mice. The combination of monoclonal antibody blocking cytotoxic T-lymphocyte-associated protein (CTLA-4) and anti-programmed cell death protein 1 (PD-1) further enhanced the ability of cGAMP to initiate immune cell infiltration. Of importance, they demonstrated that CD31^+^/VEGFR2^+^/CD45^−^ endothelial cells were the main source of type I IFN and antitumour immunity response in the murine model of melanoma and ex vivo model of human melanoma cells [117]. Tumour endothelial cells are one of the key players in modulating the immune cells in the tumour microenvironment. Moreover, endothelial cells are an important aspect of the tumour microenvironment, in terms of providing CAFs.

CAFs are a heterogeneous population of cells with different origins. Endothelial to mesenchymal transition (EndMT) is one of the sources of CAFs in tumour environments [118]. During EndMT, endothelial cells lose their markers, such as CD31, VE-cadherin, and vWF, and acquire mesenchymal markers, such as fibroblast specific protein 1 (FSP1) and alpha-smooth muscle [119]. Zeisberg et al., for the first time, showed the transition of endothelial cells to mesenchymal cells in B16F10 tumours. This was validated in *Tie2-cre*; *R26Rosa-lox-Stop-lox-LacZ* transgenic mice, where the Tie2-Cre positive endothelial cells expressed FSP1 [120]. Since the study by Zeisberg, increasing evidence has supported the endothelial plasticity and importance of EndMT in melanoma. The TGF-β pathway has been identified as one of the main inducers of EndMT in melanoma. More importantly, TGF-β1 produced by melanoma cells enhanced the number of melanoma cells adhering to endothelial cells and facilitated their trans-endothelial migration [121]. EndMT contributes to the extravasation of cancer cells and metastasis by diminishing cell–cell junctions, a key feature of the endothelial phenotype lost in EndMT. Using human melanoma-conditioned media, Platel et al., revealed the role of NAPDH oxidases (NOX) in EndMT in human umbilical cord blood endothelial cells. Melanoma cell-conditioned medium increased endothelial reactive oxygen species production via NOX1 and NOX2 and augmented cell migration [122]. More investigation on EndMT in tumour microenvironment revealed the role of two transcription factors in EndMT in melanoma, ERG, and FLI1. The combination knock-out of these transcription factors in endothelial cells induced EndMT. Melanoma tumours also display reduced expression of ERG and FLI1 in tumour endothelial cells. Moreover, lower expression of ERG in melanoma samples has been related to poor prognosis and overall survival [123]. This indicates the plasticity of endothelial cells in a pathological environment. In human melanoma patients, cellular communication network factor 2 (CCN2) is overexpressed by CAFs and negatively correlated with disease-free outcomes [124]. Moreover, CCN2 expression is correlated with the expression of angiogenic genes after gene set enrichment analysis. Depletion of CCN2 in CAFs reduced the total volume of tumour, vascular volume, and tumour vasculogenesis in the B16F10 subcutaneous tumours [124]. This provides more insights into how cell–cell interaction in the tumour microenvironment affects tumour vascularization and is correlated with patients’ outcomes. Melanoma dataset analysis revealed that female patients have a higher burden of CAFs and endothelial cells, compared to male patients. Moreover, overexpression of FGF1 and FGF2 in CAFs and endothelial cells have been observed in female patients [125]. In a recent meta-analysis of 213 uveal melanoma patient samples, it was indicated that, among immune cells, B cell infiltration is associated with a better prognosis. Conversely, CD8^+^ T cells, NK cells, macrophages M1 and M2, fibroblasts, and endothelial cells were linked with poor prognosis, and endothelial cell infiltration in the tumour microenvironment was correlated with relapse [126]. Tumour-associated macrophages may suppress the CD8^+^ T cells in uveal melanoma. Moreover, some uveal melanoma tumours possess a mutation in the BAP1 gene, which is a tumour suppressor involved in the epigenetic modulation of chromatin [127] and may suppress immune responses. It seems that uveal melanoma is inconsistent with the other tumours, in which the infiltration of inflammatory related immune cells is associated with better prognosis [128].

## 5. Role of Vascularization in Delivery of Immune Cells and Drugs

Tumour vascularization is important for the survival of cancer cells. Moreover, studies have identified mechanisms underpinning the role that tumour endothelium plays in tumour immunosuppression [129]. In the tumour environment, tumour endothelial cells express a lower level of adhesion molecules involved in leukocyte-endothelial cell interaction, such as intercellular adhesion molecule 1 (ICAM1), E-selectin, CD34, and vascular cell adhesion molecule1 (VCAM1), compared to normal endothelial cells [130]. Tumour endothelial cells promote the infiltration of regulatory T cells via the common lymphatic endothelial and vascular endothelial receptor [131]. Furthermore, localisation and activation of NK cells are mediated by the lymphocyte-associated protein LFA-1 and vascular lymphocyte function-associated protein VLA-4, as well as the recognition of ICAM1 and VCAM1 on tumour endothelial cells [132]. Angiogenic growth factors have various effects on the expression of these adhesion molecules. VEGF promotes, whereas bFGF inhibits, the adhesion of leukocytes via ICAM1 and VCAM1 to the tumour endothelial cells [132]. Melanoma tumours reduce the leukocyte-vessel wall interaction by reducing endothelial adhesion molecules [133]. In the absence of TNF-α, bFGF reduces the expression of ICAM1 in the tumour-associated endothelial cells and adhesion of leukocytes to endothelial cells [133]. High endothelial venules (HEVs), usually restricted to secondary lymphoid organs, have been reported in solid tumours, such as melanoma. Endothelial cells lining HEVs have a cuboidal morphology and express specific sets of genes that facilitate their interaction with the bloodstream, which make them distinguishable from the endothelial cells lining normal blood vessels [134]. The density of HEVs is correlated with the amount of CD3^+^ T, CD8^+^ cytotoxic T, and CD20^+^ B cells that infiltrate the tumour. This indicates the importance of HEVs in the infiltration of leukocytes into human melanoma tumours [135]. In a recent study by Asrir et al., it was demonstrated that tumours that escape the immune system had fewer tumour-associated HEVs. Immunotherapy with CTLA-4 increased the frequency (around 2%) of HEVs raising CD4^+^ and CD8^+^ T cell infiltration. Moreover, a higher number of tumour-associated HEVs in melanoma lesions that were treated with a combination of CTLA-4 and anti-PD-1 is predictive of better clinical responses and survival [136].

A key barrier to the success of drug therapy is whether it can reach the desired target tumour cells. Previously, it was mentioned that one of the hallmarks of tumour vasculature is its leaky structure, due to weak cell–cell junctions and poor pericyte coverage, as a result of the tumour microenvironment. Tumour vascular permeability augments interstitial fluid pressure (IFP) in tumours. Studies have identified IFP as one of the important factors that hinders drug and oxygen delivery [137,138]. IFP in solid tumours establishes a pressure gradient from the centre of the tumour to the periphery. This causes the discharge of fluid at the periphery, which leads to oedema, a pressure barrier that enhances the efflux of fluids from the tumour [139]. Abnormal blood vessels, high IFP, and ECM stiffness augmentation lead to enhanced permeability and retention in solid tumours. In normal tissues, molecules larger than 40 KDa cannot cross normal blood vessels; however, leaky vessels and enhanced permeability and retention allow the passage of molecules ranging from 40 to 70 KDa [140]. Many studies focus on the normalisation of tumour blood vessels for improved drug delivery [141,142]. It is believed that the normalisation of tumour vasculature repairs pericytes coverage and perfusion, reduces hypoxia and metastasis, and enhances drug delivery. In a study by Park et al., it was demonstrated, that Tie2 activation and Ang2 depletion induced tumour vessel normalisation and drug delivery in various tumours. Furthermore, tumour normalisation altered the tumour microenvironment and augmented immune cell infiltration [143]. A recent study investigated the role of matrix metalloproteinase in tumour endothelial cells. MMP14 depletion in endothelial cells reduced vessel permeability and metastasis by increasing pericyte coverage [144]. Stabilising the VE-cadherin and endothelial junctions enhanced normalisation and changed leukocyte trafficking. RNA sequencing data demonstrated overexpression of VE-cadherin in endothelial cells regulates chemokines, such as CCL2 and CXCL10, which are involved in CD8^+^ T cells infiltration [145]. Imbalanced pro- and anti-angiogenic factors lead to leaky and abnormal vessels. However, it remains unclear if vessel normalisation is enough to improve patient outcomes. Studies suggest dose-dependent anti-angiogenic treatment can balance the pro- and anti-angiogenic factors and have beneficial effects through this mechanism [146].

## 6. Targeting the Tumour Vascularization with Antiangiogenic Drugs

**Figure 3:**
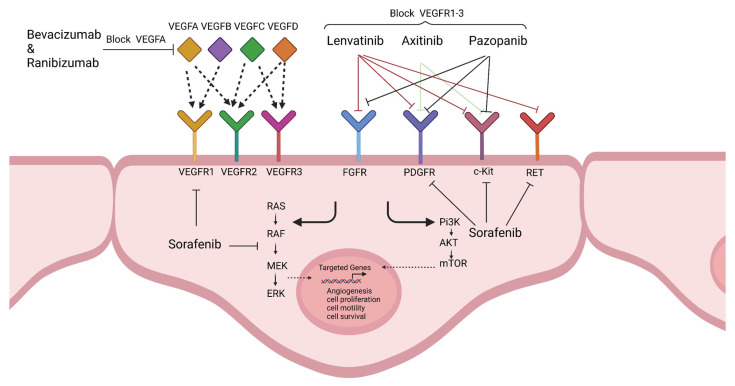
Mode of action of FDA-approved anti-angiogenic drugs used in melanoma clinical trials. Bevacizumab is the monoclonal antibody against VEGFA, and it inhibits the interaction of VEGFA with VEGFR1 and VEGFR2. Ranibizumab is a recombinant monoclonal antibody against VEGFA with similar action as Bevacizumab. Sorafenib inhibits kinases that target VEGFR1-3, PDGFRβ, RET, c-Kit, RAF-1, and BRAF. Lenvatinib is a multiple kinase inhibitor of VEGFR1-3, PDGFR, FGFR, RET, and c-Kit. Axitinib inhibits the VEGFR1-3, PDGFR, and c-Kit, and Pazopanib blocks the VEGFR1-3, c-Kit, PDGFR, and FGFR. Created with BioRender.com.

The concept of antiangiogenic therapy was initiated in the 1970s to hinder tumour vascularization and growth [15]. Folkman proposed that anti-angiogenic drugs inhibit the secretion of angiogenic factors by cancer cells [147]. During the early stages of treatment with anti-angiogenic drugs, vascular normalisation, reduction in hypoxia, and better oxygen perfusion have been observed [148]. With anti-angiogenic therapy, the phenotype of tumour endothelial cells can be reversed, and the expression of adhesion molecules in tumour vasculature upregulated. This augments the leukocyte-endothelial cell interaction and immune cell infiltration in melanoma tumours [149]. One of the first anti-angiogenic drugs was IFNα/β, derived from human leukocytes and human fibroblasts [150]. One of the major pathways that has been targeted by anti-angiogenic drugs is the VEGF pathway. In 1993, the anti-VEGF monoclonal antibody inhibited the growth of rhabdomyosarcoma, glioblastoma, and leiomyosarcoma tumours in vivo [151]. In the 1990s, Folkman and his colleagues discovered a diverse range of anti-angiogenic factors. An analogue of Fumagillin was found to inhibit the proliferation of endothelial cells, without cell apoptosis [152]. Bevacizumab (Avastin), a VEGFA inhibitory monoclonal antibody, initiated the era of anti-angiogenic therapy in 2004 for metastatic colorectal cancer [153]. Bevacizumab prevents the activation of the VEGF pathway by binding to VEGFA and preventing the activation of VEGFR1 and VEGFR2 [154]. Other anti-angiogenic drugs are tyrosine kinase inhibitors, sunitinib, sorafenib, and pazopanib, which inhibit VEGFRs, PDGFR, c-Kit, and RET (Figure 3). Some anti-angiogenic drugs in clinical trials for melanoma have been listed in Table 1.

Several preclinical and clinical studies have investigated the effect of anti-angiogenic drugs as a single agent for patients with different types and stages of melanoma [164,165]. Bevacizumab is well-tolerated and reduces metastasis in a subset of patients [166]. In one study, adjuvant bevacizumab after melanoma tumour resection improved disease-free survival interval but did not affect the overall survival. The better overall survival was positively correlated with BRAF mutation [167]. Targeting VEGF signalling and tyrosine kinase receptors alone is not sufficient to block vessel formation or induce vessel regression. In melanoma tumours, pericytes have been shown to protect the vascular network against anti-angiogenic drugs. Analysis of human melanoma metastases samples from patients with adjuvant treatment with bevacizumab and murine melanoma tumours treated with anti-VEGF demonstrated vascular network maturation [168]. This observation in both tumour models was accompanied by augmented vessel diameter and normalisation of vessels, vessel maturation, and, ultimately, resistance to anti-VEGF therapy [168]. Sorafenib blocks the phosphorylation of tyrosine kinase receptors and inhibits the MAPK signalling pathway, as well as the downstream Raf kinase isoforms [169]. Sorafenib has been used for the treatment of advanced renal cell carcinoma, hepatocellular carcinoma, and thyroid cancer [170]; however, as a single agent [171,172] or in combination with chemotherapy drugs [163], it has little or no anti-tumour activity in advanced melanoma patients. Overall, most clinical trials of FDA-approved anti-angiogenic drugs have had a limited impact on disease-free intervals and no statistically significant effect on the overall survival of patients.

Metastatic melanoma patients benefited more from the combination of anti-angiogenic drugs with chemotherapy drugs, such as carboplatin or paclitaxel [158], or in combination with ICIs [173]. The combination of anti-angiogenic drugs with ICIs could potentially represent a promising strategy and increase the activity of ICIs [174]. Preclinical and clinical studies suggest that immune suppression driven by tumour vascularization can lead to resistance to ICIs. Analysis of somatic mutations and transcriptomes of pre-treatment melanoma patients’ biopsies with PD-1 blockade (pembrolizumab and nivolumab) identified pro-angiogenic factors associated with resistance to ICIs in the non-responding patients, compared to responding pre-treatment tumours [175]. A recent phase I clinical trial by Hodi and colleagues investigated the effect of combining a monoclonal antibody against angiopoietin 2 (MEDI3617) with CTLA-4 in patients that had histologically confirmed unresectable or metastatic melanoma. They observed an alteration in the circulating CD8^+^ T cells, inducible T cell costimulator, and human leukocyte antigen DR^+^ CD4^+^ [176]. Patients with advanced mucosal melanoma respond poorly to anti-PD-1 monotherapy, especially in Asian and Caucasian populations [177]. At 3 years, the survival update demonstrated that the combination of toripalimab, an anti-PD-1, with axitinib, a tyrosine kinase inhibitor, improved the overall survival [177]. RNA sequencing and expression profiling of patients’ samples from the dual therapy trial showed a combination of angiogenesis and immune-related genes. Patients with these changes in gene expression had a better progression-free survival [177]. These studies highlight that combinations of anti-angiogenic drugs and ICIs improve the overall survival rates in advanced melanoma patients, where ICIs alone may fail.

## 7. Conclusions

Tumour vascular formation has been shown to play an important role in tumour growth, extravasation, and, subsequently, metastatic spread. Various modes of vascularization have been observed in melanoma tumours, and most studies have focused on the role of endothelial cells and their contribution to angiogenesis. EPCs are a population of cells with the capacity to proliferate, transdifferentiate, and migrate to the tumours, in response to cancer cell signals. Thus, vasculogenesis facilitated by EPCs represents a novel therapeutic target, as it potentially drives mesenchymal transition and, vessel immaturity. Various preclinical studies have reported EPCs selectively migrating into melanoma tumours [102,178], and recent preclinical studies have uncovered that anti-angiogenic drugs may miss tissue-resident EPCs and, in turn, the neovascular formation characteristics they exert [102]. It is imperative that researchers consider this mode of vascular network formation and how these cells may contribute to abnormal vessel formation across many tumour subtypes. Future studies from here will require investigation of molecular underpinnings and how tissue-resident EPCs contribute to melanoma vascularization at different stages of disease progression, leading the way for drug intervention.

## Figures and Tables

**Table 1:** List of anti-angiogenic drugs for melanoma.

Anti-Angiogenic Agent	Mode of Action/TARGET	Type of Melanoma	Clinical Indications	Reference
Axitinib (Inlyta^®^)	Tyrosine kinase inhibitor of VEGFR1, -2,-3, c-Kit, and PDGFR [155]	Human mucosal melanoma; advanced BRAF wild-type melanoma	Combination with toripalimab; combination with paclitaxel/carboplatin	[156,157]
Bevacizumab (Avastin^®^)	Monoclonal antibody to VEGFA	Human mucosal melanoma, metastatic melanoma	Combination with carboplatin plus paclitaxel	[158]
Lenvatinib mesylate (Lenvima^®^)	Kinase inhibitor against VEGFR1, -2, -3, PDGFR, RET, FGFR, c-Kit	Melanoma	Combination with pembrolizumab	[159]
Pazopanib (Votrient^®^)	Tyrosine kinase inhibitor of VEGFR1, -2, -3, PDGFR, FGFR, and c-Kit	Metastatic melanoma	Combination with paclitaxel	[160]
Ranibizumab (Lucentis^®^)	Monoclonal antibody to VEGFA	Uveal melanoma	As a single agent	[161]
Sorafenib (Nexavar^®^)	Kinase inhibitor against VEGFR, PDGFR, BRAF, RAF-1, c-Kit, and RET	Metastatic melanoma	As a single agent and in combination with bortezomib, carboplatin, and paclitaxel	[162,163]

## Abbreviations

ANG2	Angiopoietin 2
BMP	Bone morphogenic protein
CAFs	Cancer-associated Fibroblast
CCN2	Cellular communication network factor 2
CTLA-4	Cytotoxic T-lymphocyte-associated 4
DLL4	Delta-like ligand 4
ECFCs	Endothelial colony-forming cells
ECM	Extra cellular matrix
EndMT	Endothelial to mesenchymal transition
EPCs	Endothelial progenitor cells
eNOS	Endothelial nitric oxide synthesis
EVMM	Extravascular migratory metastasis
EVP	Endovascular progenitor cells
FGFs	Fibroblast growth factors
FSP1	Fibroblast specific protein 1
HEVs	High endothelial venules
HMVECs	Human microvascular endothelial cells
ICAM1	Intercellular adhesion molecule 1
ICIs	Immune checkpoint inhibitors
IFI	Interstitial fluid pressure
IFN	Interferon
MMPs	Matrix metalloproteinases
MMPs	Matrix metalloproteases
NK cells	Natural killer cells
NICD	Notch intracellular domain
PD-1	Programmed cell death protein 1
RBPJ	Recombination signal-binding protein for immunoglobulin kappa J
TSP1	Thrombospondin 1
uPAR	Urokinase-type plasminogen activator receptor
VCAM1	Vascular cell adhesion molecule 1
VE-cadherin	Vascular endothelial cadherin
VEGF	Vascular endothelial growth factor
VEGFR	Vascular endothelial growth factor receptor
vWF	von Willebrand factor
